# Bayesian shared parameter joint models for heterogeneous populations

**DOI:** 10.1007/s11222-025-10647-1

**Published:** 2025-06-12

**Authors:** Sida Chen, Danilo Alvares, Marco Palma, Jessica K. Barrett

**Affiliations:** MRC Biostatistics Unit, University of Cambridge, Cambridge, CB2 0SR Cambridgeshire UK

**Keywords:** Bayesian inference, Clustering, Joint model, Longitudinal data

## Abstract

Joint models (JMs) for longitudinal and time-to-event data are an important class of biostatistical models in health and medical research. When the study population consists of heterogeneous subgroups, standard JMs may be inadequate, leading to misleading results or loss of information. Joint latent class models (JLCMs) and their variants have been proposed to incorporate latent class structures into JMs. JLCMs are useful for identifying latent subgroups, uncovering deeper insights into relationships between the outcomes, and improving prediction performance. We consider the problem of Bayesian inference for the generic form of JLCMs, which poses significant computational challenges due to the complex nature of the posterior distribution. We propose a new Bayesian inference framework to tackle these challenges. Our approach leverages state-of-the-art Markov chain Monte Carlo techniques and parallel computing for parameter estimation and model selection regarding the number of latent classes. Through a simulation study, we demonstrate the feasibility and superiority of our proposed method over the existing approach. Additionally, we provide practical guidance on model and prior specification, which has received little attention, to facilitate the implementation of such complex models. We illustrate our method using data from the PAQUID prospective cohort study, where the outcomes of interest include a longitudinal measurement of cognitive performance and time to dementia diagnosis. Our analysis provides deeper insights into the latent class characteristics underlying the study population.

**Supplementary Information:**

The online version contains supplementary material available at 10.1007/s11222-025-10647-1.

## Introduction

In many clinical and epidemiological studies, repeated measurements of biological markers and time-to-event data are simultaneously collected. For instance, in cohort studies on chronic conditions such as cardiovascular disease and lung disease, there has been great interest in characterizing the association patterns between marker trajectories and key health events, such as disease progression or death (Barrett et al. 2019; Su et al. 2021). Joint models (JMs) have become an increasingly popular statistical modelling framework for jointly analyzing such longitudinal and time-to-event outcomes and demonstrate great potential for advancing medical insights and supporting patient monitoring (Rizopoulos 2012; Papageorgiou et al. 2019).

In some scenarios, such as multi-center studies, the study population may naturally consist of heterogeneous sub-populations, with each one exhibiting potentially different longitudinal and time-to-event profiles. It may also be the case that there is additional heterogeneity that cannot be well explained by the recorded variables. In these situations, the standard JM may no longer be appropriate, and a naive implementation could lead to misleading inference results or loss of information. Joint latent class models (JLCMs) arise as an alternative framework to the standard JM, which explicitly model the latent subgroup structure by adopting a mixture model approach (see Proust-Lima et al., 2014 for an overview). A fundamental assumption underlying the basic JLCM is the conditional independence of the longitudinal and time-to-event outcomes given the latent class membership. In other words, this implies that the association between the two outcome processes is fully explained via the shared latent class. While this assumption greatly alleviate the computational burden in model estimation, it no longer offers straightforward interpretation on the association structure between the longitudinal and time-to-event outcomes, which would be of interest in many clinical studies. Additionally, violation of this assumption could potentially necessitate the use of a larger number of latent classes, making them less interpretable. Therefore, the basic JLCM is mostly useful for prediction problems.

To improve modelling flexibility and interpretability, the basic JLCM has been extended in more recent works in order to introduce additional dependency between the outcomes within each class. This is achieved by sharing random effects across the longitudinal and time-to-event submodels, and we refer to the resulting models as shared parameter JLCMs (see, e.g., Liu et al., 2015, 2020; Andrinopoulou et al., 2020; Wong et al., 2022). These models, however, introduce new challenges to statistical inference due to the presence of high-dimensional discrete and continuous latent variables. In frequentist methods, estimation is performed by maximizing the marginal likelihood function with all latent variables integrated out. For shared parameter JLCMs, this can be computationally prohibitive due to the need for numerical approximations to handle the integrals over random effects, and optimization can be challenging due to the presence of multiple local maxima. For Bayesian methods, inference is based on the posterior distribution of model parameters, along with the latent variables, and numerical integration with respect to random effects is not required. Available information on the data can be incorporated via priors, which can also have a regularizing effect on inference. In this paper, we focus on the Bayesian paradigm due to its appeal from both modelling and computational perspectives. To our knowledge, the only existing Bayesian method is that proposed by Andrinopoulou et al. (2020), who introduced a two-stage estimation framework. In stage 1, an overfitted mixture (a mixture with potentially more classes than necessary) is employed to infer the number of latent classes by removing ‘empty’ classes. In stage 2, conditional on the results from stage 1, a shared parameter JLCM is refitted. However, there are several limitations to this approach. One challenge with the overfitted mixture model is that it can be computationally difficult to fit due to its complexity. Additionally, the number of latent classes inferred is highly sensitive to a threshold parameter, which is set in a relatively ad hoc manner. Andrinopoulou et al. (2020) used classical Markov chain Monte Carlo (MCMC) methods, implemented in JAGS, for posterior sampling. However, with high-dimensional and multimodal posterior spaces, such as in this model, these MCMC methods may suffer from poor mixing and convergence issues. Another important issue not properly addressed in their work is the presence of multimodality in the posterior, even with label ordering constraints. This issue can be particularly significant when using standard noninformative priors, as is commonly done in JMs. In such scenarios, relying on a single MCMC chain can lead to biased inference.

In this paper, we propose a new Bayesian inference framework for shared parameter JLCMs, addressing some of the key limitations of existing Bayesian approaches. Our method is built on a finite mixture model framework. We consider models with plausible numbers of latent classes. A more appropriate model for the given data can then be selected through Bayesian model comparison, which may be conducted using suitable information criteria. To perform posterior inference for a given model, differently from Andrinopoulou et al. (2020), we utilize the No-U-Turn Sampler (NUTS), an auto-tuned Hamiltonian Monte Carlo algorithm, for sampling all continuous parameters (Hoffman et al. 2014). NUTS is particularly advantageous when the posterior is of high dimensionality and complexity, and is widely regarded as the state-of-the-art MCMC method (Štrumbelj et al. 2024). To appropriately handle the multimodal posterior, we propose a computationally simple yet effective approach that involves embarrassingly parallel MCMC sampling, leveraging multi-core computing. The effectiveness of our proposed method, and its superiority over the existing approach, is demonstrated through a simulation study that follows and extends the settings considered in Andrinopoulou et al. (2020). Besides computational developments, we provide practical guidance on model and prior specification, which have received little attention yet are highly relevant to the practical implementation of such models. For instance, we found that allowing for covariate-dependent mixture weights can be important for guarding against estimation bias in certain parameters, while appropriate prior elicitation for variance parameters can improve model identifiability and MCMC convergence. We illustrate our proposed method using data from the well-known PAQUID prospective cohort study (Letenneur et al. 1994), accessed via the lcmm R package (Proust-Lima et al. 2023). We re-examine the latent subgroup structure underlying the population, guided by the longitudinal outcome of cognitive aging and time to dementia diagnosis, and provide deeper insights into the characteristics of the latent subgroups.

The rest of this paper is structured as follows. In Section 2, we present the formulation of the shared parameter JLCM. Section 3 discusses several key aspects related to the Bayesian estimation of the joint model. In Section 4, we present a simulation study to compare our method with the existing approach and investigate the impact of a specific model misspecification. An application to data from the PAQUID cohort study is presented in Section 5. Finally, Section 6 concludes with a discussion of the proposed method and potential future perspectives.

## Shared parameter joint latent class models

In this section we present the model in its general form. We assume the population of sample size *n* consists of a fixed *G* latent classes, where *G* may be unknown. Each latent class is characterized by a class-specific joint distribution of the longitudinal and time-to-event outcomes formed via shared random effects. The model consists of the following three submodel components.

### The class membership submodel

Let $c_{i}$ denote the latent class membership indicator for individual *i*, $i=1,\ldots ,n$, with $c_{i}=g$, $g=1,\ldots , G$, if subject *i* belongs to the *g*th class. The indicator $c_{i}$ is unobserved and is modelled using a categorical distribution with a probability vector $\pi _{i} = (\pi _{i1},\ldots ,\pi _{iG})$, where $\pi _{ig}=P(c_{i}=g)$. The membership probabilities are typically related to a set of *p*-dimensional time-independent baseline covariates $W_{i}$, via the softmax link function

1 $$\begin{aligned} \pi _{ig} &  = P(c_{i}=g \mid W_{i}) \nonumber \\ &  = \frac{\exp (\psi _{g0}+W_{i}^{T}\psi _{g})}{\sum _{k=1}^{G}\exp (\psi _{k0}+W_{i}^{T}\psi _{k})}, \quad g=1,\ldots ,G, \end{aligned}$$

where $\psi _{g}=(\psi _{g1},\ldots ,\psi _{gp})$ are the class-specific vectors of regression coefficients associated with $W_{i}$. For identifiability purpose, we set $\psi _{G0}$ and $\psi _{G}$ to zeros. When no external covariates are considered, the membership probability vector becomes the same across all subjects, i.e., $\pi _{i}=\pi$ for all *i*. In this scenario, Andrinopoulou et al. (2020) proposed an alternative model, where $\pi$ is directly modelled on the simplex using a Dirichlet distribution. However, with this approach, incorporating covariates is difficult, and as we will show in Section 4, the omission of relevant covariates can be problematic as it can introduce estimation bias to other model parameters.

### The longitudinal submodel

Let $y_{i}(t)$ denote the value of the longitudinal outcome of subject *i* measured at time *t*, and let $y_{i}=(y_{i1},\ldots ,y_{in_{i}})$ be the observed $n_{i}$-dimensional longitudinal response vector, where $y_{ij}=y_{i}(t_{ij})$, $j=1,\ldots ,n_{i}$. Conditional on the latent class *g*, the longitudinal outcome is typically modelled using a generalized linear mixed model (GLMM) framework. An important difference from the standard JM ($G=1$) is that we now need to introduce class-specific random effects $b_{ig}$ to characterize the deviation of a subject’s marker trajectory relative to the average marker trajectory of class *g*. Assuming the same set of random effects across classes (i.e., $b_{i1}=b_{i2}=\ldots =b_{iG}$) can, therefore, be problematic and may lead to biased inference. We model the *r*-dimensional random effects vector $b_{ig}$ using a multivariate normal distribution $N(0,\Sigma _{g})$, where $\Sigma _{g}$ is assumed to be diagonal for computational convenience. Given $c_{i}=g$ and $b_{i}$, where $b_{i}=(b_{i1},b_{i2},\ldots ,b_{iG})$, the response $y_{ij}$ are assumed to be independent and belong to a member of the exponential family with density

2 $$\begin{aligned} &  f(y_{ij} \mid c_{i}=g, b_i) \nonumber \\ &  \quad = \exp \left\{ \frac{y_{ij} \zeta _{ij}(b_{ig}) - a_{1}(\zeta _{ij}(b_{ig}))}{a_{2}(\eta _{g})} - a_{3}(y_{ij}; \eta _{g})\right\} , \end{aligned}$$

where $\zeta _{ij}(b_{ig})$ and $\eta _{g}$ denote the natural and dispersion parameters in the exponential family, respectively, and $a_1(\cdot )$, $a_2(\cdot )$, and $a_3(\cdot )$ are known functions specifying the member of the exponential family. The conditional mean of $y_{ij}$ given the class membership and random effects is related to the linear predictors via

3 $$\begin{aligned} &  E[y_{ij}\mid c_{i}=g, b_{i}, X_i(t), Z_i(t)]\nonumber \\ &  \quad =a_{1}'(\zeta _{ij}) =g^{-1}(X^{T}_{i}(t_{ij})\beta _{g}+Z^{T}_{i}(t_{ij})b_{ig}), \end{aligned}$$

where $a_{1}'(\cdot )$ denotes the first derivative with respect to its argument, $g(\cdot )$ denotes a known monotonic link function specified according to the member of the exponential family, $\beta _{g}$ is a vector of class-specific regression coefficients, and $X^{T}_{i}(t)$ and $Z^{T}_{i}(t)$ denote the possibly time-dependent design vectors for the fixed and random effects, respectively. In the case of continuous longitudinal response, the GLMM reduces to a standard Gaussian linear mixed model (LMM):

4 $$\begin{aligned} (y_{ij} \mid c_{i}=g, b_{i}) \sim N(\mu _{i}(t_{ij} \mid c_{i}=g, b_{i}, X_i(t), Z_i(t)), \sigma ^{2}_{g}), \end{aligned}$$

where $\mu _{i}(t_{ij} \mid c_{i}=g, b_{i}, X_i(t), Z_i(t))=X^{T}_{i}(t_{ij})\beta _{g}+Z^{T}_{i}(t_{ij})b_{ig}$ and $\sigma ^{2}_{g}$ is the class-specific error variance.

### The time-to-event submodel

Let $T^{*}_{i}$ be the true event time of interest and $C_{i}$ be the censoring time for subject *i*. We define the observed event time as $T_{i}=\min (T^{*}_{i}, C_{i})$, and the censoring indicator variable $\Delta _{i}=I(T^{*}_{i}\le C_{i})$, where $I(\cdot )$ is the indicator function. Conditional on the latent class membership, we model the risk of the event via the class-specific hazard function as

5 $$\begin{aligned}&h_{i}(t \mid c_{i}=g, b_i, \tilde{W}_{i})\nonumber \\&\quad =h_{0g}(t)\exp \left\{ \tilde{W}_{i}^{T}\gamma _{g}+f(\beta _{g},b_{ig},t,\alpha _{g})\right\} , \end{aligned}$$

where $h_{0g}(t)$ is the baseline hazard function parameterized by $\phi _g$, $\gamma _{g}$ denotes the vector of regression coefficients associated with the vector of exogenous risk factors $\tilde{W}_{i}$, and $f(\cdot )$ serves the role to link the longitudinal and the time-to-event processes within a latent class. In this paper, for illustration purpose, we consider the so-called current value association structure, where $f(\cdot )=\alpha _{g}\mu _{i}(t_{ij} \mid c_{i}=g, b_{i}, X_i, Z_i)$, with $\alpha _{g}$ characterizing the strength of the association. This is the most popular option in the JM literature but other reasonable functional forms may be considered, depending on the application context. Note that without the inclusion of the function $f(\cdot )$, (5) reduces to a standard proportional hazard model and the resulting full model becomes the basic JLCM of Proust-Lima et al. (2014).

## Bayesian inference

### The Bayesian model

Let the full dataset $D^{(n)}=(D_{1},\ldots ,D_{n})$, where $D_{i}=(y_{i},T_{i},\Delta _{i})$, $c=(c_{1},\ldots ,c_{n})$, $\varvec{b}=(b_{1},\ldots ,b_{n})$, and let $\Theta =(\{\psi _{g0}\}_{g=1}^{G-1},\{\psi _g\}_{g=1}^{G-1}, \{\eta _g\}_{g=1}^{G}, \{\beta _g\}_{g=1}^{G}, \{\phi _g\}_{g=1}^{G}, \{\alpha _g\}_{g=1}^{G}, \{\gamma _g\}_{g=1}^{G}, \{\Sigma _g\}_{g=1}^{G})$ denote the collection of all parameters associated with the model presented in Section 2. We assume that the joint posterior density of model parameters, random effects and latent class indicator variables, takes the form

6 $$\begin{aligned} &  p(\Theta , b, c \mid D^{(n)}) \propto \nonumber \\ &  \quad \prod _{i=1}^{n} \left( p(D_i \mid b_i, c_i, \Theta ) p(c_i \mid \Theta ) \prod _{g=1}^{G} p(b_{ig} \mid \Theta )\right) \times p(\Theta )\nonumber \\ &  \quad = \prod _{i=1}^{n} \left( \prod _{g=1}^{G} \Big ( p(y_i \mid b_i, c_i = g, \Theta ) \right. \nonumber \\ &  \qquad \left. p(T_i, \Delta _i \mid b_i, c_i = g, \Theta ) \pi _{ig} \Big )^{I(c_i=g)} p(b_{ig} \mid \Theta ) \right) \nonumber \\ &  \qquad \times p(\Theta ), \end{aligned}$$

where $p(y_i \mid b_i, c_i = g, \Theta )$ is given by (2) and (3), $\pi _{ig}$ is given by (1), and $p(b_{ig} \mid \Theta )$ is the density of multivariate normal distribution with mean 0 and covariance matrix $\Sigma _g$. $p(T_i, \Delta _i \mid b_i, c_i = g, \Theta )$ is the likelihood contribution from the survival submodel, which takes the form

7 $$\begin{aligned} &  p(T_i, \Delta _i \mid b_i, c_i = g, \Theta ) \nonumber \\ &  \quad = h_{i}(t \mid c_{i}=g, b_i)^{I(\Delta _i=1)}\nonumber \\ &  \quad \times \exp \left\{ -\int _{0}^{T_i}h_{i}(s \mid c_{i}=g, b_i)ds\right\} , \end{aligned}$$

where the hazard function $h_i$ is given by (5). The integral in (7) cannot be computed analytically in general and numerical approximation is required. In our implementation, we used the Gaussian-Legendre quadrature method (with 15 quadrature points). We assume that the joint prior density $p(\Theta )$ factorises into a product of marginal prior densities for each parameter, some of which require careful consideration, as elaborated below. Overly strong priors can have an undesirable influence on inference — such as on the selection of the number of latent classes (see Section 3.4) — especially when the sample size is small. Conversely, vague prior settings may lead to a highly multimodal posterior, and MCMC may suffer from convergence issues. To help with model identifiability and MCMC sampling, we propose to use mildly informative priors where possible. Hyperparameters can be selected based on available information for the data and expected structure for the resulting model, and/or guided by an empirical Bayes approach.

Among the model parameters, the priors for variance parameters in our longitudinal submodel require special attention, given their important contribution to shaping the latent class structure, particularly when survival patterns do not strongly distinguish between classes. For the residual error variance $\sigma _{g}^{2}$, we propose using light-tailed priors to anchor its value within plausible within-subject noise levels, as inflated $\sigma _{g}^{2}$ would blur class boundaries in the longitudinal data, thereby hindering the identification of latent classes. In this paper, we consider the popular half-Normal distribution as a prior for $\sigma _{g}^{2}$; however, other reasonable choices with similar properties can also be used. The choice of the prior hyperparameter can be guided by the maximum likelihood estimate obtained from fitting an LMM (with $G=1$) to the longitudinal data alone, since $\sigma _{g}^{2}$ is not expected to be influenced much by the time-to-event data or by varying values of *G*.

For the random effects covariance matrices $\Sigma _g$, specifying the prior amounts to setting priors for the random effect variances. Note that despite assuming a diagonal covariance structure, the random effects can still be correlated *a posteriori* if supported by the data, as no independence constraint is imposed on their posterior distribution. In our model, the random effect variances have strong relevance for mixture component estimation, as they determine the extent of within-class heterogeneity in the longitudinal process. Inspections of model structure and empirical results from our simulations suggest that, with noninformative priors on $\Sigma _g$, the posterior can favour extremely small variances for some classes, which tend to be associated with the formation of ‘small’ clusters or with mixing issues due to instability or ill-conditioning in the posterior geometry, or excessively large variances, which often coincide with the formation of ‘large’ classes that conflict with the latent classes’ role in explaining population heterogeneity. These two extremes tend to co-occur within a single model due to compensation effects between classes. Therefore, to improve latent class interpretability and sampling, it is desirable for the prior to constrain the random effect variances within a plausible range of values. In this paper, we propose a novel prior for $\Sigma _g$ with the following structure:

8 $$\begin{aligned} p(\Sigma _{g})\propto \prod _{i=1}^{r} \text {Ga}(\Sigma _{g,ii}\mid \alpha _b,\alpha _b)\times \exp \big (-\frac{\lambda }{\sqrt{\sum _{i=1}^{r}\Sigma _{g,ii}}}\big ), \nonumber \\ \end{aligned}$$

where $\text {Ga}(\cdot \mid \alpha ,\beta )$ denotes a Gamma density with shape and rate parameters equal to $\alpha _b$ and $\Sigma _{g,ii}$ denotes the *i*th diagonal entry of $\Sigma _g$. To our knowledge, this prior structure has not been used in existing joint latent class models or similar hierarchical mixture models. It is easy to show that (8) defines a proper joint prior for the random effect variances $\Sigma _{g,ii}$, and that it has a unique mode lying on the line $\Sigma _{g,11}=\Sigma _{g,22}=\cdots =\Sigma _{g,rr}$. The independent Gamma priors aim to reflect the belief about the plausible range of variances, while the additional exponential term penalises joint degeneracy, i.e., the variances simultaneously becoming too small. The effect of this penalty term vanishes as the variances move away from the origin, or as the regularising parameter $\lambda$ approaches zero. We found that using a $\lambda > 0$ is particularly helpful for larger values of *G*. Our prior construction is loosely motivated by the penalised complexity priors (Simpson et al. 2017), but applies penalisation in the opposite direction: specifically, our prior penalises model degeneracy, rather than shrinkage toward a simpler base model. To get an idea of the plausible range of random effects variances for a specific model, we can fit a basic JLCM conditional on different candidate values of *G* using the MLE approach developed in Proust-Lima et al. (2017), implemented via the lcmm R package, noting that random effects variances are generally expected to decrease as *G* increases. The penalising parameter $\lambda$ can be chosen so that the mode of the resulting joint prior (which can be easily found using an optimiser) is close to the mode of the Gamma prior. In our experience, results are generally robust, provided the hyperparameters are chosen in a reasonable way as explained above. Later, in Sections 4 and 5, we provide concrete examples and explanations regarding the choice of priors.

### Posterior sampling

The resulting posterior distribution defined via equation (6) is not analytically tractable so we resort to MCMC methods for posterior inference. We consider the NUTS algorithm due to its efficiency for sampling from high dimensional parameter spaces. NUTS requires the underlying density to be smooth because it relies on gradient computation, so it cannot be used to directly sample from (6) due to the presence of discrete variables. Instead, we use NUTS to sample from the posterior with $c_i$ marginalized out from (6), $p(\Theta , b \mid D^{(n)})$, which is proportional to

9 $$\begin{aligned}&\prod _{i=1}^{n} \left[ \left( \sum _{g=1}^{G} p(y_i \mid b_i, c_i = g, \Theta ) p(T_i, \Delta _i \mid b_i, c_i = g, \Theta ) \pi _{ig} \right) \right. \nonumber \\&\quad \left. \prod _{g=1}^{G}p(b_{ig} \mid \Theta ) \right] \times p(\Theta ). \end{aligned}$$

In equation (9), we implicitly assume a centered parameterization for the random effects. Depending on the informativeness of the data, a non-centered parameterization (i.e., modelling random effects as scaled standard normals rather than directly from their marginal distributions) may offer better mixing of NUTS; see, e.g., Betancourt and Girolami (2015) for related discussions.

Posterior samples of latent class allocation variable $c_i$ can be obtained by sampling exactly from its full conditional distribution $p(c_i\mid \Theta ^{(j)}, b^{(j)}, D^{(n)})$, which is a categorical distribution with weights for class *g*, $g=1,\ldots ,G$, proportional to

10 $$\begin{aligned} p(y_i \mid b_i^{(j)}, c_i = g, \Theta ^{(j)}) p(T_i, \Delta _i \mid b_i^{(j)}, c_i = g, \Theta ^{(j)}) \pi _{ig}^{(j)}, \nonumber \\ \end{aligned}$$

where $b_i^{(j)}$ and $\Theta ^{(j)}$ are the *j*th MCMC samples from $p(\Theta , b \mid D^{(n)})$, and $\pi _{ig}^{(j)}$ is computed using $\Theta ^{(j)}$. Note that based on the sampled $c_i$, the posterior class membership probability can be estimated as

11 $$\begin{aligned}&\hat{p}(c_i=g \mid D^{(n)})=\frac{1}{T}\sum _{j=1}^{T}I(c_{i}^{(j)}=g), \nonumber \\&i=1,\ldots ,n,\quad g=1,\ldots ,G. \end{aligned}$$

The class membership for each subject can then be estimated based on the maximum *a posteriori* (MAP) decision rule, i.e., $\hat{c}_{i}=\text {argmax}_{g} \hat{p}(c_i=g \mid D^{(n)})$.

### Tackling multimodality in the posterior

For mixture-type models, an obvious cause of multimodality in the posterior is the invariance of the likelihood under permutations of the latent class labels. This can create problems for MCMC-based inference because label switching may occur (in theory) during MCMC runs, rendering the marginal posterior for class-specific parameters unidentifiable. To tackle this issue, some form of ordering constraint or post-processing of MCMC samples is typically required; see, e.g., Stephens (2000). However, in complex hierarchical models like the one considered here, label switching is highly unlikely within a practical number of MCMC runs, as it would require a simultaneous jump in all class-specific parameters. In our experiments, label switching was not observed. What needs to be addressed is that multiple well-separated local regions of high posterior density can arise even within a fixed class labelling. This is observed in MCMC runs (particularly with noninformative priors) and is overlooked in Andrinopoulou et al. (2020). This makes single-chain MCMC inference problematic, as the chain may get trapped in a local region depending on the initialization.

Various MCMC schemes have been proposed in the literature to handle complex multimodal distributions, notably parallel tempering, which involves running multiple chains that communicate with each other. However, effective use of such algorithms requires careful design and tuning, and they can be computationally prohibitive for complex models. Our idea here is motivated by Yao et al. (2022), who propose a Bayesian stacking approach to take advantage of fully parallel MCMC sampling, aiming to combine locally stuck chains to enhance prediction performance. When the focus is on inference, a modification leads to the following approach:

1. Run *M* parallel chains with different initializations.
2. Clustering the *M* parallel chains into *K* clusters, defined over regions $\Omega _1, \ldots , \Omega _K$, with $K \le M$.
3. Select a region $\Omega _k$ with probability proportional to
  $$ w_k = \int P(D \mid b, \varvec{\Theta }) P(b \mid \varvec{\Theta }) P(\varvec{\Theta }) \mathbb {I} \left( (b, \varvec{\Theta }) \in \Omega _k \right) dbd\varvec{\Theta }, $$
  and then draw a sample with replacement from $\Omega _k$.

In step 2, a between-chain mixing measure such as $\hat{R}$ can be used for clustering the chains (Vehtari et al. 2021), and the weights $w_k$ can be estimated by any valid Monte Carlo estimator for the marginal likelihood based on samples in $\Omega _k$ (Llorente et al. 2023). Assuming that the chains cover all important regions of the support, the final samples would form approximate samples from the target posterior, as this is essentially a importance resampling procedure (Smith and Gelfand 1992). Note that this parallel sampling strategy is designed to address multimodal posteriors for inferential purposes, which is fundamentally different from standard applications of multiple-chain MCMC, where the goal is typically either convergence diagnostics or increasing sample size by aggregating chains. In the multimodal setting, standard diagnostics may fail to detect issues, as a small number of chains may become trapped in the same local mode, and naively combining samples from chains targeting different modes can lead to nonsensical results.

When the dimensionality of the parameter space becomes high, the clustering procedure in step 2 can still be challenging to implement. To improve computational feasibility, in our implementation, we consider a simplified version of the approach above with the clustering step removed. We then select one among the *M* convergent chains that yields the highest weight, upon which inference is based. This approach can be justified under the assumption that the posterior is dominated by a single region, $\Omega _k^{*}$, in terms of posterior mass — which may be reasonable with the use of mildly informative priors — or if we are willing to base inference on the mode that yields the largest probability given the data. In our simulations, we found this simplified scheme performed satisfactorily. To estimate the weights $w_k$, we consider the truncated harmonic mean estimator (Robert and Wraith 2009; Chen and Finkenstädt 2024), which is given by

12 $$\begin{aligned} \left( \frac{1}{T} \sum _{i=1}^{T} \frac{h\left( b^{(i)}, \varvec{\Theta }^{(i)}\right) }{p\left( D^{(n)} \mid b^{(i)}, \varvec{\Theta }^{(i)}\right) p\left( b^{(i)}, \varvec{\Theta }^{(i)}\right) } \right) ^{-1}, \end{aligned}$$

where $b^{(i)}$ and $\varvec{\Theta }^{(i)}$ are the *i*th MCMC samples out of a total of *T* samples. A convenient choice for *h* is

13 $$\begin{aligned} h(b,\varvec{\Theta }) = \frac{1}{V(\epsilon ) \beta T} \sum _{j: (b^{(j)},\varvec{\Theta }^{(j)}) \in \mathcal {H}_{\beta }} \mathbb {I}(d((b^{(j)},\varvec{\Theta }^{(j)}), (b, \varvec{\Theta })) < \epsilon ), \nonumber \\ \end{aligned}$$

where $V(\epsilon )$ is the volume of the ball centered at $(b,\varvec{\Theta })$ with radius $\epsilon$ (small), $\mathcal {H}_{\beta }=\{(b^{(j)},\varvec{\Theta }^{(j)}): p(D^{(n)} \mid b^{(i)}, \varvec{\Theta }^{(i)}) p(b^{(i)}, \varvec{\Theta }^{(i)})>q_\beta \}$, and $q_\beta$ is the empirical upper $\beta$ quantile of $p(D^{(n)} \mid b^{(i)}, \varvec{\Theta }^{(i)}) p(b^{(i)}, \varvec{\Theta }^{(i)})$. When the radius is sufficiently small, $h(b^{(i)}, \varvec{\Theta }^{(i)})$ will take the value of $1/(V(\epsilon ) \beta T)$ if the sample is from $\mathcal {H}_{\beta }$, and zero otherwise. $V(\epsilon )$ does not need to be computed, as it will cancel out during normalization. It is worth noting that the posterior within the selected region, $\Omega _k$, may still have a complex structure. It is therefore important that the MCMC algorithm efficiently explores this local region, which motivates our use of the NUTS algorithm. To further aid convergence, we use MLEs to initialise part of the parameters in each chain. More specifically, for the residual error variances, fixed effect parameters, and baseline hazard parameters ($\sigma _g, \beta _g, \phi _g, \gamma _g$), we first obtain MLEs from the lcmm R package and then apply random, independent perturbations to each parameter. All other parameters, including random effects, are randomly initialised from independent Gaussian or uniform distributions. Our initialisation strategy therefore introduces sufficient randomness across chain-specific initial values to increase the chance of exploring distinct posterior regions beyond the (local) MLE neighbourhood.

### Selection of number of classes

In many application contexts, the number of latent classes *G*, if any, is unknown. To infer this value, we choose not to adopt the overfitted mixture approach proposed by Andrinopoulou et al. (2020) due to the aforementioned difficulties. Instead, we propose simultaneously considering models with candidate values of *G*, treating it as a model comparison problem. One popular class of methods for Bayesian model choice is based on marginal likelihood or Bayes factors, which generally have consistency in model selection when the true generating process is among the candidate models, though it is worth noting that, in practice, seeking the ‘true’ number of classes may not be meaningful or realistic. An important issue with these approaches is that the results can be sensitive to the choice of priors. For our model, we find that the prior for the random effect variances can have a strong influence on the estimated marginal likelihood. The Bayesian information criterion (BIC) is popular choice for approximating the marginal likelihood without reliance on prior specifications (Schwarz 1978); however, its theoretical justification does not hold for singular models, which typically arise in hierarchical and mixture settings (Watanabe 2010). Moreover, it is computationally challenging to compute in our setting due to the need to integrate out random effects.

Here, we consider a different class of methods to guide our choice on *G*, which are based on expected predictive error or generalization loss. The two most popular criteria for this purpose are the Bayesian leave-one-out information criterion (LOOIC) and the widely applicable information criterion (WAIC) (Watanabe 2010; Gelman et al. 2014; Vehtari et al. 2017). Both criteria are fully Bayesian and possess desirable theoretical properties, such as invariance to reparameterisation and applicability to singular models, and are computationally cheaper to estimate than standard K-fold cross-validation. LOOIC targets $\sum _{i=1}^{n}\log p(D_i \mid D_{-i})$, where in our context $p(D_i \mid D_{-i})=\int p(D_i \mid \varvec{b}, \varvec{c}, \Theta )p( \varvec{b}, \varvec{c}, \Theta \mid D_{-i})d\varvec{b}d\varvec{c}d\Theta$. WAIC, on the other hand, targets $\sum _{i=1}^{n}\log p(D_i \mid D^{(n)})-p_{\text {WAIC}}$, where $p(D_i \mid D^{(n)})=\int p(D_i \mid \varvec{b}, \varvec{c}, \Theta )p( \varvec{b}, \varvec{c}, \Theta \mid D^{(n)})d\varvec{b}d\varvec{c}d\Theta$, and $p_{\text {WAIC}}$ is defined as the sum of the posterior variance of the log predictive density for each data point. Asymptotically, the two criteria are equivalent. A higher LOOIC or WAIC (or lower, if multiplied by $-2$ to be on the deviance scale) thus indicates a better predictive ability of the model.

To infer the number of latent classes using LOOIC or WAIC, we consider the following forward selection procedure that is in line with the parsimony principle. We define a candidate set of models with $G = 1$ to $G_{\text {max}}$. Starting from the simplest model with $G=1$, we iteratively compare the current best model to all more complex alternatives. A more complex model is selected only if it (i) demonstrates significantly better predictive performance, and (ii) has a higher number of ‘effective classes’, which we define below. The procedure terminates once the current best model has been compared to the most complex candidate. For (i), the significance of the difference in predictive performance can be examined by comparing the z-score, which is computed by dividing the estimated difference in LOOIC or WAIC by the estimated standard error associated with the difference, to the critical value for a one-tailed test based on the standard normal distribution at a given significance level (Sivula et al. 2020). In our experience (see also results in Section 4), we found that a relatively stringent significance level, such as $\alpha =0.1\%$ is desirable to guard against the risk of overfitting. For (ii), the effective class size is defined as the number of classes whose estimated posterior proportion exceeds a specified (small) threshold. This arises from the observation that when models are overfitted, some estimated classes may be effectively empty or occupy only a tiny proportion of the sample — which is essentially a simpler model. Such automatic regularization has long been known as a desirable property of Bayesian inference. The choice of the threshold for the class proportion depends somewhat on the sample size and the application context. In our simulations, we found that a small threshold greater than 0, such as around 1%, works well. Regarding the choice of $G_{\text {max}}$, in the absence of prior knowledge, it can be set based on an initial guess (e.g., informed by sample size). $G_{\text {max}}$ can then be increased dynamically until LOOIC or WAIC no longer improve, or until empty or near-empty classes begin to appear — both of which may be viewed as signs of overfitting. We want to emphasize that in practice, one should not blindly follow results from such an automatic procedure, as they can be influenced by deviations in model formulation from the unknown true data-generating process (if one exists) and the quality of the estimates of these criteria. Therefore, these results should always be considered with cautiousness in conjunction with model interpretation and the study context.

## Simulation study

We consider a simulation study consisting of two simulation settings. Setting I follows and extends from the simulation study considered in Andrinopoulou et al. (2020), with the aim of providing a ‘sanity check’ for the proposed method and evaluating the performance of our approach in comparison to theirs. Setting II, modified from Setting I, aims to evaluate the impact of the misspecification of the class membership submodel on inference.

### Simulation settings

#### Setting I

In this setting, we consider the same scenarios with $G=1$ to $G=3$ as in Andrinopoulou et al. (2020), and include an additional, more challenging case with $G=4$. In all four scenarios, the total sample size is fixed at $n=900$, and the latent class membership is randomly assigned to each individual according to pre-specified class proportions (i.e., the class memberships are independent of covariates). The class-specific longitudinal trajectory is generated from an LMM specified as

14 $$\begin{aligned} y_i(t) = \beta _{g0} + \beta _{g1} t + b_{ig0} + b_{ig1} t + \beta _{g2} \text {male}_i + \epsilon _{ig}(t), \end{aligned}$$

where $\epsilon _{ig}(t)\sim N(0, \sigma ^{2}_{g})$, $(b_{ig0}, b_{ig1})\sim N(0,\Sigma _g)$, and $\text {male}_i$ is an artificial covariate for gender, simulated from a Bernoulli distribution with a probability 0.5 for being male (labelled as 1). The class-specific time-to-event model is specified as

15 $$\begin{aligned} h_i(t) = \phi _{g0} t^{\phi _{g0} - 1} \exp \left\{ \phi _{g1} + \gamma _{g1} \text {age}_i + \alpha _g \mu _i(t) \right\} , \end{aligned}$$

where $\mu _i(t)=\beta _{g0} + \beta _{g1} t + b_{ig0} + b_{ig1} t + \beta _{g2} \text {male}_i$. $\text {age}_i$ is generated from a normal distribution centered at 45 with a standard deviation of 15.70. For each individual, a random censoring time $C_i$ is generated from a uniform distribution between zero and 17.5. We refer to Table 1 of Andrinopoulou et al. (2020) (for $G=1$ to 3) and Table 1 in the supplementary material (for $G=4$) for further details on the parameter value settings. For each scenario, we evaluated the performance of our proposed approach as described in Section 3.4 in selecting the number of classes. We considered candidate values of *G* ranging from one to four (for the $G=1$ to 3 scenarios) or five (for the $G=4$ scenario), and evaluated three different significance levels ($\alpha =5\%$, $1\%$, $0.1\%$) in combination with three different class proportion thresholds ($0\%$, $1\%$, $2\%$). We also implemented the approach of Andrinopoulou et al. (2020) using their code for comparison in the new scenario with $G=4$. Conditional on the true value of *G*, we further examine the performance of the proposed algorithm in terms of accuracy in parameter estimation and classification. Under Setting I, the estimation model (for a given *G*) assumes the same model formulation as used in the generating process.

#### Setting II

For this setting we have fixed $G=2$. The data generation process for the longitudinal and survival submodel are the same as in Setting I. We consider 4 different scenarios to generate the class membership:

- Scenario 1: class allocation independent of any covariates (same as Setting I with $G=2$).
- Scenario 2: $W_i=\tilde{x}_i$, where $\tilde{x}_i\sim N(0,1)$, with $\psi _{10}=-0.4$, $\psi _{11}=1$.
- Scenario 3: $$W_i=(\text {male}_i, \text {age}_i)$$, with $$\psi _{10}=2$$, $$\psi _{11}=4$$, $$\psi _{12}=-0.1$$.
- Scenario 4: $$W_i=(\text {male}_i, \text {age}_i, \tilde{x}_i)$$, with $$\psi _{10}=2$$, $$\psi _{11}=4$$, $$\psi _{12}=-0.1$$, $$\psi _{13}=1$$.

For each scenario, we consider two estimation models, which represent two common choices for modelling the class membership. Model 1 assumes homogeneous class membership probabilities as used in Setting I (i.e., correctly specified for Scenario 1), whereas model 2 considers the same set of covariates as considered in class-specific JM with $W_i=(\text {male}_i, \text {age}_i)$ (i.e., correctly specified for Scenario 3). Results from the two estimation models are compared in terms of the accuracy of estimating the commonly shared parameters, as well as classification accuracy.

### Estimation settings

To implement the proposed algorithm, we use the following set of priors for the model parameters throughout:

16 $$\begin{aligned} \begin{aligned}&\beta _{gi}, \gamma _{gi}, \phi _{g1}, \alpha _g \sim N(0,5^2), \quad \psi _{gi}\sim N(0,1),\\ &\phi _{g0} \sim \text{ Gamma }(2,0.5), \quad \sigma ^{2}_{g}\sim \text{ half-Normal }(0,0.5^2), \\ &p(\Sigma _{g})\propto \prod _{i=1}^{2} \text{ Ga }(\Sigma _{g,ii}\mid 1.5,1.5)\exp \left( -\frac{1}{\sqrt{\sum _{i=1}^{2}\Sigma _{g,ii}}}\right) , \end{aligned} \end{aligned}$$

where $\text {Gamma}(\alpha ,\beta )$ represents a Gamma distribution with shape parameter $\alpha$ and rate parameter $\beta$ and half-Normal $(\mu ,\sigma ^2)$ denotes a half-Normal distribution with location parameter $\mu$ and scale parameter $\sigma$. For fixed effect and association parameters ($\beta _{gi}$, $\gamma _{gi}$, $\phi _{g1}$ and $\alpha _g$), the priors are weakly informative, similar to those used in standard JMs. We use a slightly more informative prior for the fixed effect parameters $\psi _{gi}$ in the class membership submodel to discourage the mixture weights from getting too close to the boundary of the simplex. For the Weibull shape parameter $\phi _{g0}$, the Gamma prior we use has a flat, unimodal shape and right skewness, covering plausible values in survival analysis. For the variance parameters, the prior hyperparameters are partly informed from an empirical Bayes perspective. For $\sigma ^{2}_{g}$, the scale parameter in the half-Normal prior is chosen to be close to the MLE results obtained from fitting an LMM to the longitudinal data. To set the joint prior for $\Sigma _{g}$, we specify the shape and rate parameters of the Gamma density as 1.5 (for $G > 1$), which we found to sufficiently cover plausible values of the random effects variance. $\lambda$ in the penalty term is set to one, placing the mode of the joint prior close to that of the unpenalized version. When fitting the model with $G=1$, i.e., no latent class, we use the same prior settings as in (16), except that for $\Sigma _{g,ii}$, we use an inverse Gamma prior with shape and scale parameters set to 0.01, which is a common weakly informative choice for the standard JM.

To perform posterior sampling, as described in Section 3.2, we used the Stan modelling language via the R interface provided by the rstan R package (Stan Development Team 2024), which provides a convenient implementation of the NUTS. In our simulations, we used a non-centered parameterization of the random effects, as we found it provided better mixing compared to the centered counterpart. We used the default settings for the optional tuning parameters in NUTS, as they generally worked well and achieved a good balance between sampling and computational efficiency. However, in practice, for complex models where divergent transitions or stability issues arise, these default settings can be adjusted. For example, the target acceptance rate (referred to as $\texttt {adapt\_delta}$) can be increased from the default value of 0.8 to a larger value (such as 0.9 or higher) to improve the robustness of NUTS, although higher values may substantially increase sampling time. To implement the parallel sampling scheme as described in Section 3.3, we used $M=6$ parallel chains, each run for 6000 iterations, with the first 3000 iterations discarded as burn-in. For each chain, the 3000 post-burn-in samples were further thinned by an interval of 3. These settings were found to be sufficient for our simulation; however, in more challenging scenarios, more chains, a longer burn-in period, or a larger thinning interval may be required. To estimate the weights for each chain using the truncated harmonic mean estimator, we used $\beta = 0.8$ (the results seem generally robust for different values of this parameter). To estimate the LOOIC and WAIC, and the quantities required for computing the z-score, we used the method developed in Vehtari et al. (2017), which is implemented in the loo R package (Vehtari et al. 2024). All computations were performed on the Cambridge Service for Data Driven Discovery (CSD3) High-Performance Computing (HPC) system using the Ice Lake CPUs.

### Results

**Fig. 1:**
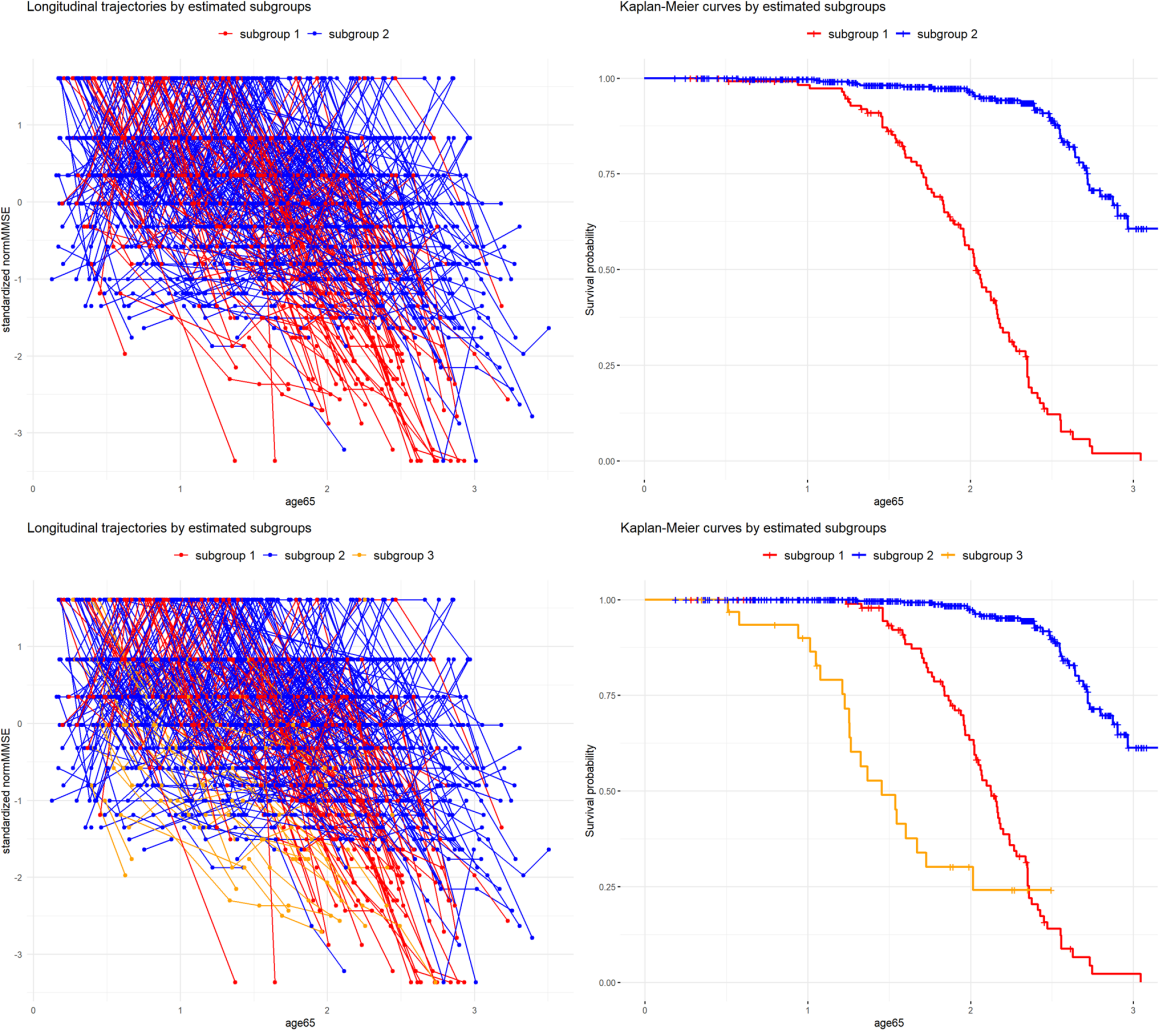
Trajectories of standardized normMMSE and Kaplan-Meier curves by estimated subgroups (indicated by colours). The top panel displays results for $G=2$, and the bottom panel for $G=3$

Table 1 summarizes the results for model selection under simulation Setting I obtained using the procedure described in Section 3.4, when the class proportion threshold is set to $1\%$. Results for the other two threshold choices are shown in Tables 2 and 3 in the supplementary material. We can see that using a class proportion threshold greater than 0 significantly improves model selection accuracy, with $2\%$ yielding the highest accuracy among the choices considered. For a given threshold, LOOIC consistently outperforms WAIC, and as the significance level becomes more stringent, the performance of both criteria improves. These results demonstrate the value of our proposed approach, rather than simply selecting the model based on the value of LOOIC or WAIC alone. Indeed, both criteria tend to stabilise once the number of latent classes reaches the true value, with a slight tendency to overfit (see Figures 1 and 2 in the supplementary material). In particular, they are not effective at penalising more complex models when additional small clusters are present. When compared to the approach of Andrinopoulou et al. (2020), our method demonstrate superior accuracy and stability across all four scenarios (especially when class proportion threshold is greater than 0). For $G=1$ to 3, Andrinopoulou et al. (2020) achieved classification accuracies of $30\%$, $49\%$ and $72\%$, respectively, conditional on their best-performing tuning parameter $\psi =15\%$ (see Table 3 of Andrinopoulou et al., 2020 for details). For the most challenging scenario with $G=4$, $\psi =15\%$ yields an accuracy of only $7\%$, while other choices of $\psi$ give accuracies of $1.5\%$, $50\%$, and $48.5\%$ for $\psi =1\%$, $5\%$, and $10\%$, respectively.

Our proposed algorithm demonstrates satisfactory performance in both point and interval estimation. Tables 5 (under Model 1) and 4 in the supplementary material present the estimation results conditional on $G=2$ and $G=3$, respectively. With regard to classification accuracy, defined as the proportion of correctly classified subjects based on the MAP decision rule with class allocation probabilities computed as described in Section 3.2, we achieve consistently high accuracy. The median and interquartile range are $98.8\% \ (98.6\%, 99.0\%)$ for $G=2$, $99.4\% \ (99.2\%, 99.6\%)$ for $G=3$, and $96.3\% \ (95.9\%, 96.7\%)$ for $G=4$, respectively.

**Table 1:** Performance of the proposed model selection approach using LOOIC and WAIC under simulation Setting I. The threshold for effective class size is set to $1\%$. $z=1.65$, 2.33, and 3.09 are critical values for the z-score corresponding to one-tailed tests at significance levels of $5\%$, $1\%$, and $0.1\%$, respectively. For each significance level, the true number of classes ($\%$) was computed as the proportion of times the correct number of classes was inferred, based on 200, 200, 197, and 71 replications of data for $G=1$, 2, 3, and 4, respectively. For $G=3$ and 4, results for all data replications could not be obtained due to some MCMC runs exceeding the computational time limits of the HPC system

**Scenario**	**Criteria**	**True number of classes (%)**		
		$z=1.65\ (\alpha =5\%)$	$z=2.33\ (\alpha =1\%)$	$z=3.09\ (\alpha =0.1\%)$
**G = 1**	LOOIC	91.0	92.0	93.5
	WAIC	88.5	89.5	90.5
**G = 2**	LOOIC	95.5	95.5	97.0
	WAIC	93.5	94.0	95.0
**G = 3**	LOOIC	91.3	92.3	94.4
	WAIC	88.8	89.8	94.4
**G = 4**	LOOIC	90.1	91.5	94.4
	WAIC	85.9	90.1	90.1

**Table 2:** Results for simulation Setting II (Scenario 3). For each parameter, bias and standard deviation (SD) are evaluated based on the posterior mean obtained from 200 data replications, and coverage is the proportion of times the 95% credible interval contains the true parameter value across the 200 replications. Classification accuracy is summarized using the median and interquartile range (shown in brackets), with accuracy for each replication calculated as the proportion of correctly classified subjects. Model 1: model with homogeneous mixture weights (ignoring covariate effects on the class membership). Model 2: model with covariate dependent mixture weights (use gender and age only)

**Parameter**	**True Value**	**Model 1**			**Model 2**		
		Bias	SD	Coverage (%)	Bias	SD	Coverage (%)
$\beta _{1,0}$	8.03	-0.002	0.144	93.0	0.000	0.141	94.0
$\beta _{1,1}$	-0.16	0.002	0.014	93.5	0.000	0.011	94.5
$\beta _{1,2}$	-5.86	-0.090	0.206	84.5	-0.008	0.143	95.0
$\beta _{2,0}$	-8.03	0.010	0.034	95.5	0.002	0.034	94.5
$\beta _{2,1}$	0.46	-0.064	0.077	83.0	0.000	0.071	94.0
$\beta _{2,2}$	12.2	-0.152	0.161	52.5	-0.008	0.073	95.5
$\gamma _{1,0}$	-4.85	-0.332	0.472	87.0	-0.052	0.420	92.5
$\gamma _{1,1}$	-0.02	-0.002	0.010	91.5	-0.001	0.006	92.5
$\gamma _{2,0}$	-4.85	-0.173	0.278	88.5	-0.029	0.281	94.5
$\gamma _{2,1}$	0.09	0.002	0.005	92.0	0.000	0.005	94.0
$\alpha _{1}$	0.38	0.019	0.047	91.0	0.003	0.034	92.5
$\alpha _{2}$	0.08	-0.010	0.020	81.5	0.000	0.009	96.0
$\xi _{1}$	1.8	0.120	0.138	85.5	0.031	0.118	96.0
$\xi _{2}$	1.4	0.003	0.066	92.5	0.010	0.058	94.5
$\sigma ^{2}_{1}$	0.4761	0.000	0.015	94.5	0.001	0.015	94.0
$\sigma ^{2}_{2}$	0.4761	-0.008	0.023	95.0	-0.011	0.022	95.0
$\Sigma _{1,11}$	0.87	-0.083	0.071	80.5	-0.003	0.071	95.0
$\Sigma _{1,22}$	0.02	0.002	0.028	95.5	0.001	0.002	95.5
$\Sigma _{2,11}$	0.02	0.041	0.034	75.5	0.024	0.018	91.0
$\Sigma _{2,22}$	0.91	-0.018	0.101	93.5	-0.002	0.098	95.5
		Classification accuracy			Classification accuracy		
		97.4 (97.0, 97.9)			99.1 (98.9, 99.3)

**Table 3:** Results for simulation Setting II (Scenario 4). The settings are the same as those in Table 2

**Parameter**	**True Value**	**Model 1**			**Model 2**		
		Bias	SD	Coverage (%)	Bias	SD	Coverage (%)
$\beta _{1,0}$	8.03	$-$0.023	0.127	93.5	$-$0.025	0.127	95.5
$\beta _{1,1}$	$-$0.16	0.001	0.010	94.5	0.000	0.010	96.0
$\beta _{1,2}$	$-$5.86	$-$0.072	0.145	86.5	0.022	0.145	93.5
$\beta _{2,0}$	$-$8.03	0.011	0.032	93.0	0.004	0.032	94.0
$\beta _{2,1}$	0.46	$-$0.061	0.077	81.5	$-$0.002	0.078	91.0
$\beta _{2,2}$	12.2	$-$0.135	0.083	50.0	$-$0.014	0.071	96.0
$\gamma _{1,0}$	$-$4.85	$-$0.373	0.439	86.0	$-$0.081	0.397	93.5
$\gamma _{1,1}$	$-$0.02	$-$0.001	0.007	94.5	$-$0.001	0.006	94.5
$\gamma _{2,0}$	$-$4.85	$-$0.177	0.265	91.0	$-$0.056	0.268	95.0
$\gamma _{2,1}$	0.09	0.002	0.005	93.5	0.001	0.005	94.0
$\alpha _{1}$	0.38	0.025	0.035	87.5	0.005	0.032	96.0
$\alpha _{2}$	0.08	$-$0.007	0.009	84.5	0.000	0.009	96.5
$\xi _{1}$	1.8	0.129	0.134	81.0	0.038	0.123	94.0
$\xi _{2}$	1.4	0.005	0.052	93.5	0.010	0.052	95.0
$\sigma ^{2}_{1}$	0.4761	0.000	0.014	95.5	0.001	0.014	93.5
$\sigma ^{2}_{2}$	0.4761	$-$0.007	0.025	93.0	$-$0.010	0.024	94.5
$\Sigma _{1,11}$	0.87	$-$0.061	0.071	88.0	0.013	0.076	94.5
$\Sigma _{1,22}$	0.02	0.000	0.002	93.5	0.001	0.002	95.0
$\Sigma _{2,11}$	0.02	0.042	0.024	69.5	0.026	0.018	89.0
$\Sigma _{2,22}$	0.91	$-$0.008	0.085	97.5	0.007	0.090	97.5
		Classification accuracy			Classification accuracy		
		97.4 (97.1, 97.9)			99.0 (98.8, 99.2)		

For simulation Setting II, Tables 2 and 3 summarise the estimation results for Scenarios 3 and 4, respectively, while results for Scenarios 1 and 2 are presented in Tables 5 and 6 in the supplementary material. Model 1, which ignores covariates in class membership submodel, can incur significant bias and much lower coverage for certain parameters when the true class allocation depends on covariates that are also related to the longitudinal and time-to-event processes (see Tables 2 and 3). Model 2, which uses the same covariates as those considered in the class-specific JM, provides consistently satisfactory performance regardless of the ground truth, offering much better estimation accuracy in Scenarios 3 and 4 (see Tables 2 and 3) and similar accuracy in Scenarios 1 and 2 (see Tables 5 and 6 in the supplementary material) compared to Model 1. Note that the classification performance appears to be minimally impacted by these specifications. Although not shown here, we conducted additional analyses under various settings. We considered an alternative version of Model 1, where the softmax parametrisation for the class membership submodel is replaced by a Dirichlet model on the homogeneous mixture weights, with the concentration parameter set to 1. The results are nearly identical, and the key patterns remaining unchanged. When *G* is not fixed, we found that ignoring relevant covariates in the membership submodel also impacts the selection of the number of classes. In particular, we found that this omission can be compensated for by selecting additional classes. Clearly, the extent of the bias incurred and the impact on inference depend on the strength of the association between the covariates and the class allocation and longitudinal processes. In general, we would expect the impact to increase as the strength of the association grows.

## Application to the PAQUID dataset

We illustrate our proposed method using the PAQUID dataset available from the lcmm R package, which consists of a random subsample of 500 subjects from the prospective cohort study of the same name, aimed at investigating the relationship between risk factors and cognitive and functional aging diseases in the elderly population in France. The study participants were aged 65 and over at entry and were drawn from two regions in southwestern France. Information on baseline socio-demographic variables, cognitive performance, and health and medical history were recorded at an individual level over a maximum follow up period of 20 years. For more background information on the original data, we refer to Proust-Lima et al. (2017) and the references therein. Proust-Lima et al. (2017) used the basic JLCM to model and analyse the trajectory of repeated measures of a normalized version of the Mini-Mental State Examination score (normMMSE) with age and the associated risk of dementia. For their analysis, one subject was removed ($n=499$) due to the event of interest occurring earlier than the entry time. The resulting dataset contains a total of 2213 longitudinal measurements, with a median of 4 and an interquartile range of (2, 7) per individual, and $74.3\%$ of subjects had censored event times. Using the basic JLCM, Proust-Lima et al. (2017) performed model selection on the number of latent classes using the BIC, and $G=4$ latent classes were suggested. Interestingly, with $G=4$, a hypothesis test performed therein indicated that the conditional independence of the longitudinal and time-to-event outcomes, given the latent class, was not supported by the data.

Here, motivated by the analysis conducted in Proust-Lima et al. (2017), we use the shared parameter JLCM to re-analyse the same data to explore any changes in the latent class structure and to investigate the relationship between normMMSE and the risk of dementia within a latent class, which remains unexplored in earlier works. The basic setup for the longitudinal and time-to-event submodels mostly follows the JLCM used in Proust-Lima et al. (2017). To simplify prior specification and improve sampling efficiency, we further standardised the normMMSE (which ranges from 0 to 100) to have a mean of 0 and a standard deviation of 1. The longitudinal trajectory for the standardized normMMSE, conditional on the class membership *k*, is modelled via an LMM as

17 $$\begin{aligned}&(y_{ij} \mid c_i = g) = \beta _{g0} + \beta _{g1} \, age65_{ij} + \beta _{g2} \, age65_{ij}^2 + b_{ig0}\nonumber \\&\quad + b_{ig1} \, age65_{ij} + b_{ig2} \, age65_{ij}^2 + \beta _{g,CEP} \, CEP_i + \epsilon _{igj}, \nonumber \\ \end{aligned}$$

where $age65_{ij} = (age_{ij} - 65) / 10$, with $age_{ij}$ being the age of individual *i* at the *j*th visit, and $CEP_i$ is a binary variable for education, where 1 indicates the subject graduated with a primary school diploma, and 0 otherwise. We further assumed that $(b_{ig0},b_{ig1},b_{ig2})\sim N(0,\Sigma _g)$, with $\Sigma _g$ being diagonal, and that $\epsilon _{igj}\sim N(0,\sigma _{g}^{2})$. For the class-specific time-to-event submodel, we used the same time scale (i.e., $age65_{ij}$) as for the longitudinal process, and we link the time-to-event process with the longitudinal process using a current value association. The hazard function is specified as

18 $$\begin{aligned} \begin{aligned}&h_i(t \mid c_i = g) \\&\quad = h_{0g}(t) \exp ( \gamma _{g,CEP} CEP_i + \gamma _{g,male} male_i\\&\qquad + \gamma _{g,CEP \times male} (CEP_i \times male_i) + \alpha _g \mu _{ig}(t)), \end{aligned} \end{aligned}$$

where $h_{0g}(t) = \phi _{g0} t^{\phi _{g0} - 1} \exp (\phi _{g1})$, $male_i$ is a binary variable, with 1 for male and 0 for female, and $\mu _{ig}(t)$ is the conditional mean of $y_{i}(t)$ given $c_i=g$. The possible interaction effect between education and gender was not explored in the previous analysis by Proust-Lima et al. (2017), so we include it here to assess whether such an effect exists. We also note that Proust-Lima et al. (2017) did not incorporate covariates into the class membership submodel. In our analysis, however, motivated by our findings from the simulation study, we chose to include both $CEP_i$ and $male_i$ to guard against the possibility of incurring any bias on the estimation results. The membership submodel is therefore specified as

19 $$\begin{aligned} &  \pi _{ig} = P(c_{i}=g) \nonumber \\ &  \quad = \frac{\exp (\psi _{g0}+\psi _{g,CEP}CEP_i +\psi _{g,male}male_i)}{\sum _{k=1}^{G}\exp (\psi _{k0}+\psi _{k,CEP}CEP_i +\psi _{k,male}male_i)},\nonumber \\ &  \quad g=1,\ldots ,G, \end{aligned}$$

where $\psi _{G0}$, $\psi _{G,CEP}$ and $\psi _{G,male}$ are set to zeros. To complete the Bayesian model, we choose the following set of prior distributions:

20 $$\begin{aligned} \begin{gathered} \beta _{g\cdot }\sim N(0,2^2), \quad \gamma _{g\cdot }, \phi _{g1}, \alpha _g \sim N(0,3^2), \\ \psi _{g\cdot } \sim N(0,1), \quad \phi _{g0} \sim \text{ Gamma }(2,0.5),\\ \sigma ^{2}_{g}\sim \text{ half-Normal }(0,0.2^2), \\ p(\Sigma _{g})\propto \prod _{i=1}^{3} \text{ Ga }(\Sigma _{g,ii}|1.5,1.5) \\ \qquad \times \exp \left( -\frac{1}{\sqrt{\sum _{i=1}^{3}\Sigma _{g,ii}}}\right) . \end{gathered} \end{aligned}$$

Note that we used generally more informative normal priors for the fixed effect parameters than in the simulation study, partly due to the smaller sample size here, and we also accounted for the fact that the longitudinal data had been standardized. The hyperparameters for the priors on the variance parameters were informed by the MLE results obtained from the lcmm R package, as was done in the simulation study.

We first examine the number of latent classes using the approach described in Section 3.4, considering candidate values of *G* ranging from one to four due to the relatively small sample size. Posterior inference was based on $M = 6$ parallel chains, each with 12000 iterations for $G=1,\ 2,\ 3$ or 14000 iterations for $G=4$. The first 4000 (for $G=1, 2, 3$) or 6000 (for $G=4$) iterations were discarded as burn-in, based on pilot runs and convergence diagnostics. For each chain, the 8000 post burn-in samples were further thinned by an interval of 4. All other estimation settings are the same as in the simulation study. Using the most stringent significance level considered ($\alpha = 0.1\%$) and a class proportion threshold of $2\%$, as motivated by the small sample size and findings from the simulation study, $G = 3$ was selected with both LOOIC and WAIC. Table 4 shows the estimated LOOIC and WAIC for different values of *G* (on the deviance scale), along with the associated z-scores and p-values for testing differences between models with $G = k$ and $G = k + 1$. The improvement in LOOIC and WAIC is most pronounced when increasing *G* from one to two, indicating strong support for the presence of latent class structure. Although $G=4$ achieves the smallest LOOIC and WAIC, the improvement over $G=3$ is minimal. Moreover, with $G=4$, an extra small cluster is generated with only seven individuals assigned to it. Therefore, we may be skeptical about considering this as a meaningful ‘subgroup’. Comparing models with two and three latent classes, the strength of evidence for a difference in LOOIC and WAIC is close to the predefined significance threshold, as suggested by the associated z-scores and p-values. Figure 1 displays the class-specific longitudinal trajectories and Kaplan-Meier survival curves based on the estimated models with $G=2$ (top panel) and $G=3$ (bottom panel). Class membership for each individual was assigned based on the MAP class probability, as done in the simulation study. It appears that class 1 in the model with $G=2$, which is associated with more significant declines in normMMSE and a more rapid progression of dementia, is further split into two subclasses in the model with $G=3$ (labelled as subgroups 1 and 3). This visual impression is confirmed by comparing the class allocation results obtained from $G=2$ and $G=3$. Therefore, depending on the desired level of detail, either model could be of interest. As a further comparison, we also implemented the overfitted mixture approach of Andrinopoulou et al. (2020), using their default estimation settings. With the tuning parameter set to $\psi = 15\%$, the method suggests $G = 3$, which aligns with our results. However, other choices of $\psi$ risk overfitting, with $G = 4$, 5, and 6 suggested for $\psi = 10\%$, $5\%$, and $1\%$, respectively.

Here, we present results with $G=2$ which captures the major subgroup structures within the study cohort. Based on the estimated class allocation, $76.4\%$ (381/499) of individuals are classified into class 2, the relatively lower-risk group, while the remaining belong to class 1. Table 5 shows the posterior means and $95\%$ credible intervals for key parameters of interest in the model with $G=2$. The male gender shows a significant association with class membership ($\psi _{1,male}$), where, prior to observing any longitudinal data, being male decreases the chance of being classified into the relatively higher-risk group. Additionally, there is a strong and significant association between normMMSE and dementia, conditional on latent class membership, as indicated by the magnitude of $\alpha _k$ and the fact that the associated credible intervals exclude zero. The strength of the association is similar across both subgroups — an additional analysis imposing $\alpha _1=\alpha _2$ yielded almost identical LOOIC and WAIC values. The negative relationship is expected, as higher normMMSE generally reflects better cognitive function, which is anticipated to be associated with a lower risk of cognitive diseases such as dementia. The log-hazard ratio for education, $\gamma _{g,CEP}$, is estimated to be positive for both classes and appears to be significant for class 2. While this may seem contradictory to earlier results in Proust-Lima et al. (2017) where CEP had a protective effect on dementia, it is important to note that here the effect of CEP is mediated through the longitudinal marker (MMSE) via $\mu _i(t)$. The total effect of CEP on the risk of dementia, given by $\gamma _{g,CEP} + \alpha _g \times \beta _{g,CEP} + \gamma _{g,CEP \times male} \times male_i$, remains negative. For this data, CEP and gender do not show a significant interaction effect ($\gamma _{g,CEP \times male}$). The two subgroups also exhibit different levels of within-group variability in normMMSE (measured via $\sigma _g^{2}$), with the higher-risk group, class 2, showing greater within-class variability. This aligns with the clinical literature, which links neurodegenerative disorders to increased intraindividual variability in cognitive performance metrics (Hultsch et al. 2000; Gorus et al. 2008).

Our analysis so far has been conditional on assuming the current value association structure. To assess the impact of using a different association structure on the estimation results, we consider an alternative formulation using the popular current slope association, that is,

21 $$\begin{aligned} \begin{aligned}&h_i(t \mid c_i = g) \\&\quad = h_{0g}(t) \exp ( \gamma _{g,CEP} CEP_i + \gamma _{g,male} male_i \\&\qquad + \gamma _{g,CEP \times male} (CEP_i \times male_i) + \alpha _g \mu '_{ig}(t)), \end{aligned} \end{aligned}$$

where $\mu '_{ig}(t)$ is the derivative of $\mu _{ig}(t)$. The remainder of the model and prior specifications remain unchanged. We re-examined the number of latent classes using the same estimation settings as in the earlier analysis based on the current value association. With a significance level of $\alpha = 0.1\%$ and a class proportion threshold of $2\%$ as considered earlier, $G = 2$ was selected with both LOOIC and WAIC. Additional results comparing models with different candidate values of *G* are provided in Table 7 of the supplementary material. A closer examination of the estimation results for $G = 2$ (see Table 8 and Figure 3 in the supplementary material) reveals a highly significant, negative association between the current slope and the risk of the event, with the strength of association varying across the two classes. The negative relationship indicates that a quicker decline in normMMSE is associated with a higher risk of dementia, which is clinically reasonable. In addition, the key features of the estimated latent classes are similar to those obtained under the current value association, and the two models exhibit comparable predictive performance, as indicated by the nearly identical LOOIC and WAIC. Our additional analysis reaffirms that additional and interpretable dependency between the longitudinal and time-to-event processes remains, beyond what is explained by the latent class structure, and supports that $G = 2$ yields a relatively stable clustering structure in this study population. A more thorough investigation and comparison of results under different association structures, however, is beyond the scope of this paper.

**Table 4:** Results for comparing models with candidate values of *G*. For each $G=k$, LOOIC and WAIC (on the deviance scale) are computed as described in Section 3.4. The $Z_{LOOIC}$ and $Z_{WAIC}$ represent the z-scores associated with the differences in LOOIC and WAIC, respectively, comparing the models with $G=k$ and $G=k+1$. ${P_{LOOIC}}$ and ${P_{WAIC}}$ are the one-tailed p-values associated with $Z_{LOOIC}$ and $Z_{WAIC}$, respectively. $G_{eff}$ is the effective class size defined in Section 3.4, with a class proportion threshold set to $2\%$

	LOOIC	$Z_{LOOIC}$	$P_{LOOIC}$	WAIC	$Z_{WAIC}$	$P_{WAIC}$	$G_{eff}$
G = 1	4317	7.05	0	4079	7.48	0	1
G = 2	4091	3.19	0.001	3840	2.49	0.006	2
G = 3	4032	0.63	0.264	3795	0.79	0.214	3
G = 4	4018	-	-	3773	-	-	3

**Table 5:** Estimation results for the shared parameter JLCM with $G=2$. Posterior means are reported as point estimates (the corresponding posterior distributions are approximately symmetric). The 95% credible intervals (CI) are based on the 2.5th and 97.5th percentiles of the posterior samples

**Submodel**	**Parameters**	**Estimates (Posterior Mean (95% CI))**	
		Class 1	Class 2
Longitudinal	$\beta _{g,CEP}$	0.368 (-0.008, 0.718)	0.841 (0.657, 1.041)
	$\sigma ^{2}_{g}$	0.318 (0.267, 0.372)	0.224 (0.191, 0.253)
Time-to-event	$\alpha _{g}$	-3.615 (-4.909, -2.610)	-3.711 (-5.382, -2.356)
	$\gamma _{g,CEP}$	0.461 (-0.490, 1.373)	1.204 (0.015, 2.530)
	$\gamma _{g,male}$	0.148 (-1.548, 1.673)	0.342 (-1.185, 1.691)
	$\gamma _{g,CEP\times male}$	-0.044 (-1.893, 1.801)	0.926 (-1.055, 3.079)
Class membership	$\psi _{10}$	-0.532 (-1.252, 0.155)	
	$\psi _{1,CEP}$	0.199 (-0.446, 0.850)	
	$\psi _{1,male}$	-0.837 (-1.491, -0.186)	

## Discussion

In this paper, we focus on shared parameter JLCMs, which provide an important extension of the basic JLCM for study populations of a heterogeneous nature. We propose a new Bayesian inferential framework to effectively tackle the computational challenges faced by existing Bayesian methods. To enhance sampling efficiency, we adopt a strategy that enables the use of state-of-the-art MCMC methods. We effectively address potential multimodality in the posterior through a parallel sampling scheme, leveraging parallel computing power. We propose a new model selection strategy for the number of latent classes building on predictive-based criteria such as LOOIC, which are conveniently estimated from MCMC output. Through a simulation study, we demonstrate that the proposed method provides superior estimation accuracy compared to the existing approach. The feasibility of our method is further illustrated via an application to the PAQUID data, where we explored the underlying latent class structure and obtained insights into the association between the cognitive measure MMSE and the risk of dementia within each latent class.

Our results bring important insights into the practical implementation of such models. One important aspect concerns the prior specification, which has received very little attention in the context of shared parameter JLCMs. Unlike the standard JM, prior specification for JLCMs, particularly for variance parameters, requires special care. It is desirable to use relatively informative priors that exploit available knowledge, perhaps in conjunction with empirical Bayes strategies, to help with model identifiability and the convergence of the algorithm. Provided that hyperparameters are set in a reasonable way, the inference results are generally robust under our proposed prior settings. Regarding the selection of the number of latent classes, predictive criteria such as LOOIC can be informative, yet may lead to overfitting if used naively. The effective class proportion threshold and hypothesis testing procedure introduced in Section 3.4 are important safeguards against this risk. In general, a more conservative approach (i.e., using a suitably chosen small threshold and a relatively stringent significance level) seems desirable. In practice, model selection results serve as guidance only (e.g., to help shortlist candidate models), and the final choice should also take into account which model is most useful or interpretable, e.g., informed by visual and quantitative inspection of the results. We also found that including relevant covariates in the class membership model is important. While ignoring these covariates has little impact on classification accuracy, bias arises when they are related to the longitudinal and time-to-event processes. Therefore, it seems advisable to include the same set of covariates considered in class-specific JMs to mitigate potential bias. Although this work focuses on shared parameter JLCMs, we believe these considerations are relevant to more general latent class or mixture models involving multi-outcome processes.

It is possible to extend the algorithm considered here to alternative formulations of JLCMs, depending on the application context. For instance, multivariate longitudinal data can be accommodated if available, and more flexible hazard functions can be adopted when appropriate. In such more complex modelling scenarios, strategies like parameter reduction — by sharing certain parameters or assuming relationships among functionals of parameters (e.g. baseline hazards or covariance matrix of random effects) across latent classes — may help alleviate computational burden and mitigate issues related to weak identifiability. These choices can be guided by background knowledge or informed by preliminary analyses. When the focus is on prediction and multivariate longitudinal markers are present, one could consider replacing the shared parameter structure with a two-stage approach, where summaries from the raw longitudinal data are first extracted and treated as time-varying covariates in the time-to-event submodel (Alvares and Leiva-Yamaguchi 2023). A specific formulation under a discrete hazard framework using a frequentist approach has been considered in Nguyen et al. (2024). A Bayesian formulation under a more generic hazard setting may provide advantages in terms of prior regularization, prediction, and associated uncertainty quantification.

From the algorithmic perspective, it would be interesting to explore alternative approximate Bayesian inference methods that are potentially more scalable in complex, large-data settings. The MCMC-based method presented here can serve as a gold standard benchmark for evaluating these approximate algorithms. One option, motivated by the recent success of INLA for the standard JMs (Rustand et al. 2024; Alvares et al. 2024), is the possibility of adapting it to the mixture setting by combining MCMC with INLA (Gómez-Rubio and Rue 2018). In this approach, we sample from the marginal posterior of the latent class allocation variables using MCMC, and conditional on the sampled class allocation, INLA is used to fit the class-specific JM. Some preliminary experiments (not reported here) suggest that the efficiency was extremely low due to the poor acceptance rate of the MCMC step and stability issues with INLA, which arise from the need to refit the model a large number of times. A more efficient MCMC proposal and a more stable or dedicated version of the INLA program warrant further development. Variational-based methods could offer another promising option. Black-box variational algorithms, such as those provided by Stan, are not feasible for such complex models. A customized development, possibly building on recent advances in variational inference algorithms for standard JMs (Sun and Basu 2024), would be of interest.

## Supplementary Information

Below is the link to the electronic supplementary material.

Supplementary file 1 (pdf 633 KB)

## Data Availability

The PAQUID dataset used in this paper is publicly available from the lcmm R package, accessible at https://cran.r-project.org/web/packages/lcmm/. The R and Stan code used in the simulation studies and case study are available in the GitHub repository https://github.com/sidachen55/SP_JLCM.
