# Supplementary material for: Bayesian shared parameter joint models for heterogeneous populations
Source: Stat Comput. 2025 Jun 12;35(5):125. doi: 10.1007/s11222-025-10647-1 (PMC12162714; doi:10.1007/s11222-025-10647-1)
Supplement: Supplementary file 1 — (pdf 633 KB) [file 11222_2025_10647_MOESM1_ESM.pdf]

# Supplementary Material for "Bayesian shared parameter joint models for heterogeneous populations"

Sida Chen[1*], Danilo Alvares[1], Marco Palma[1], Jessica K. Barrett[1]

[1*]MRC Biostatistics Unit, University of Cambridge, Cambridge, CB2 0SR, Cambridgeshire, U.K.

*Corresponding author(s). E-mail(s): sida.chen@mrc-bsu.cam.ac.uk;
Contributing authors: danilo.alvares@mrc-bsu.cam.ac.uk;
marco.palma@mrc-bsu.cam.ac.uk; jessica.barrett@mrc-bsu.cam.ac.uk;

**Table 1**: Simulation parameters for the case with $G = 4$ latent classes. The parameter settings for classes 1 and 4 are identical to those of classes 1 and 3 in Andrinopoulou et al. (2020), respectively. The parameters for classes 2 and 3 are derived by splitting the original class 2 in Andrinopoulou et al. (2020) into two subclasses. The pre-specified class sizes are $n = 100,\ 300,\ 200,\ 300$ for classes 1 to 4, respectively.

| | $\boldsymbol{\beta_g}$ | $\sigma_g$ | diag$\{\Sigma_g\}$ | $\phi_{g0}$ | $\phi_{g1}, \gamma_{g1}$ | $\alpha$ |
|---|---|---|---|---|---|---|
| Class 1 (size $n = 100$) | (Intercept) $= 8.03$<br>Male $= -5.86$<br>Time $= -0.16$ | 0.69 | 0.87<br>0.02 | 1.8 | (Intercept) $= -4.85$<br>Age $= -0.02$ | 0.38 |
| Class 2 (size $n = 300$) | (Intercept) $= -9$<br>Male $= 10$<br>Time $= 0.5$ | 0.69 | 0.05<br>0.95 | 1.1 | (Intercept) $= -8$<br>Age $= 0.12$ | 0.1 |
| Class 3 (size $n = 200$) | (Intercept) $= -6$<br>Male $= 10$<br>Time $= 0.3$ | 0.69 | 0.2<br>0.65 | 1.6 | (Intercept) $= -1$<br>Age = -0.05 | -0.2 |
| Class 4 (size $n = 300$) | (Intercept) $= 0.03$<br>Male $= -1.96$<br>Time $= -0.01$ | 0.69 | 0.28<br>0.31 | 1.8 | (Intercept) $= 2.85$<br>Age $= -0.12$ | 0.58 |

**Table 2**: Performance of the proposed model selection approach using LOOIC and WAIC under simulation Setting I. The threshold for effective class size is set to 0 (i.e., a class with at least one subject allocated is qualified as an effective class). $z = 1.65$, 2.33, and 3.09 are critical values for the z-score corresponding to one-tailed tests at significance levels of 5%, 1%, and 0.1%, respectively. For each significance level, the true number of classes (%) was computed as the proportion of times the correct number of classes was inferred, based on 200, 200, 197, and 71 replications of data for $G = 1$, 2, 3, and 4, respectively. For $G = 3$ and 4, results for all data replications could not be obtained due to some MCMC runs exceeding the computational time limits of the HPC system.

| **Scenario** | **Criteria** | **True number of classes (%)** | | |
|---|---|---|---|---|
| | | $z = 1.65$ ($\alpha = 5\%$) | $z = 2.33$ ($\alpha = 1\%$) | $z = 3.09$ ($\alpha = 0.1\%$) |
| **G = 1** | LOOIC | 55.5 | 64.5 | 68.5 |
| | WAIC | 46.5 | 54.5 | 57.5 |
| **G = 2** | LOOIC | 50.0 | 55.0 | 63.5 |
| | WAIC | 39.0 | 44.0 | 49.5 |
| **G = 3** | LOOIC | 67.3 | 76.5 | 83.7 |
| | WAIC | 58.2 | 63.3 | 74.5 |
| **G = 4** | LOOIC | 77.5 | 80.3 | 85.9 |
| | WAIC | 63.4 | 74.6 | 80.3 |

**Table 3**: Performance of the proposed model selection approach using LOOIC and WAIC under simulation Setting I. The threshold for effective class size is set to 2%. $z = 1.65$, 2.33, and 3.09 are critical values for the z-score corresponding to one-tailed tests at significance levels of 5%, 1%, and 0.1%, respectively. For each significance level, the true number of classes (%) was computed as the proportion of times the correct number of classes was inferred, based on 200, 200, 197, and 71 replications of data for $G = 1$, 2, 3, and 4, respectively. For $G = 3$ and 4, results for all data replications could not be obtained due to some MCMC runs exceeding the computational time limits of the HPC system.

| **Scenario** | **Criteria** | **True number of classes (%)** | | |
|---|---|---|---|---|
| | | $z = 1.65$ ($\alpha = 5\%$) | $z = 2.33$ ($\alpha = 1\%$) | $z = 3.09$ ($\alpha = 0.1\%$) |
| **G = 1** | LOOIC | 94.5 | 95.5 | 96.5 |
| | WAIC | 91.0 | 92.0 | 93.5 |
| **G = 2** | LOOIC | 99.0 | 99.0 | 99.5 |
| | WAIC | 98.5 | 98.5 | 99.0 |
| **G = 3** | LOOIC | 94.9 | 95.4 | 96.4 |
| | WAIC | 93.4 | 93.9 | 96.4 |
| **G = 4** | LOOIC | 94.4 | 95.8 | 97.2 |
| | WAIC | 93.0 | 93.0 | 93.0 |

# References

Andrinopoulou, E.R., Nasserinejad, K., Szczesniak, R., Rizopoulos, D.: Integrating

**Table 4**: Results for simulation Setting I (Scenario 3) with G fixed at 3. For each parameter, bias and standard deviation (SD) are evaluated based on the posterior mean obtained from 200 data replications, and coverage is the proportion of times the 95% credible interval contains the true parameter value across the 200 replications.

| **Parameter** | **True Value** | **Bias** | **SD** | **Coverage (%)** |
|---|---|---|---|---|
| $\beta_{1,0}$ | 8.03 | 0.009 | 0.141 | 95.5 |
| $\beta_{1,1}$ | -0.16 | 0.001 | 0.023 | 95.5 |
| $\beta_{1,2}$ | -5.86 | -0.008 | 0.204 | 95.5 |
| $\beta_{2,0}$ | -8.03 | -0.004 | 0.051 | 93.5 |
| $\beta_{2,1}$ | 0.46 | 0.010 | 0.093 | 95.5 |
| $\beta_{2,2}$ | 12.2 | -0.003 | 0.073 | 94.5 |
| $\beta_{3,0}$ | 0.03 | 0.001 | 0.046 | 97.5 |
| $\beta_{3,1}$ | -0.01 | -0.001 | 0.035 | 92.5 |
| $\beta_{3,2}$ | -1.96 | 0.002 | 0.061 | 97.0 |
| $\gamma_{1,0}$ | -4.85 | -0.168 | 0.802 | 93.5 |
| $\gamma_{1,1}$ | -0.02 | -0.001 | 0.011 | 94.0 |
| $\gamma_{2,0}$ | -4.85 | -0.078 | 0.301 | 95.0 |
| $\gamma_{2,1}$ | 0.09 | 0.001 | 0.006 | 95.5 |
| $\gamma_{3,0}$ | 2.85 | 0.038 | 0.228 | 94.5 |
| $\gamma_{3,1}$ | -0.12 | -0.002 | 0.007 | 93.0 |
| $\alpha_1$ | 0.38 | 0.014 | 0.066 | 93.0 |
| $\alpha_2$ | 0.08 | 0.002 | 0.011 | 91.5 |
| $\alpha_3$ | 0.58 | 0.010 | 0.034 | 97.0 |
| $\xi_1$ | 1.8 | 0.065 | 0.228 | 95.0 |
| $\xi_2$ | 1.4 | 0.019 | 0.065 | 95.5 |
| $\xi_3$ | 1.8 | 0.032 | 0.088 | 91.5 |
| $\sigma_1^2$ | 0.4761 | 0.001 | 0.029 | 95.0 |
| $\sigma_2^2$ | 0.4761 | -0.011 | 0.029 | 93.5 |
| $\sigma_3^2$ | 0.4761 | -0.002 | 0.015 | 95.0 |
| $\Sigma_{1,11}$ | 0.87 | 0.039 | 0.164 | 95.5 |
| $\Sigma_{1,22}$ | 0.02 | 0.003 | 0.006 | 94.0 |
| $\Sigma_{2,11}$ | 0.02 | 0.031 | 0.019 | 93.0 |
| $\Sigma_{2,22}$ | 0.91 | 0.019 | 0.119 | 98.0 |
| $\Sigma_{3,11}$ | 0.28 | 0.008 | 0.037 | 92.5 |
| $\Sigma_{3,22}$ | 0.31 | 0.003 | 0.029 | 94.5 |

latent classes in the Bayesian shared parameter joint model of longitudinal and survival outcomes. Statistical Methods in Medical Research **29**(11), 3294–3307 (2020)

**Table 5**: Results for simulation Setting II (Scenario 1). For each parameter, bias and standard deviation (SD) are evaluated based on the posterior mean obtained from 200 data replications, and coverage is the proportion of times the 95% credible interval contains the true parameter value across the 200 replications. Classification accuracy is summarized using the median and interquartile range (shown in brackets), with accuracy for each replication calculated as the proportion of correctly classified subjects. Model 1: model with homogeneous mixture weights (ignoring covariate effects on the class membership). Model 2: model with covariate dependent mixture weights (use gender and age only).

| **Parameter** | **True Value** | **Model 1** | | | **Model 2** | | |
|---|---|---|---|---|---|---|---|
| | | Bias | SD | Coverage (%) | Bias | SD | Coverage (%) |
| $\beta_{1,0}$ | 8.03 | -0.006 | 0.083 | 94.0 | -0.006 | 0.083 | 94.0 |
| $\beta_{1,1}$ | -0.16 | 0.000 | 0.014 | 93.5 | 0.000 | 0.014 | 95.0 |
| $\beta_{1,2}$ | -5.86 | 0.018 | 0.123 | 96.0 | 0.018 | 0.124 | 96.5 |
| $\beta_{2,0}$ | -8.03 | 0.001 | 0.033 | 95.0 | 0.001 | 0.033 | 95.0 |
| $\beta_{2,1}$ | 0.46 | -0.006 | 0.063 | 94.5 | -0.005 | 0.063 | 95.5 |
| $\beta_{2,2}$ | 12.2 | -0.005 | 0.049 | 94.5 | -0.005 | 0.048 | 94.5 |
| $\gamma_{1,0}$ | -4.85 | -0.030 | 0.427 | 95.5 | -0.024 | 0.421 | 95.0 |
| $\gamma_{1,1}$ | -0.02 | -0.001 | 0.005 | 95.0 | -0.001 | 0.005 | 96.0 |
| $\gamma_{2,0}$ | -4.85 | -0.010 | 0.204 | 98.0 | -0.008 | 0.202 | 97.0 |
| $\gamma_{2,1}$ | 0.09 | 0.000 | 0.004 | 98.0 | 0.000 | 0.004 | 97.0 |
| $\alpha_1$ | 0.38 | 0.005 | 0.036 | 94.5 | 0.004 | 0.036 | 94.0 |
| $\alpha_2$ | 0.08 | 0.000 | 0.007 | 95.5 | 0.000 | 0.007 | 96.0 |
| $\xi_1$ | 1.8 | 0.031 | 0.125 | 92.0 | 0.028 | 0.124 | 92.5 |
| $\xi_2$ | 1.4 | 0.005 | 0.047 | 95.0 | 0.004 | 0.047 | 96.0 |
| $\sigma_1^2$ | 0.4761 | -0.001 | 0.019 | 94.0 | -0.001 | 0.019 | 92.5 |
| $\sigma_2^2$ | 0.4761 | -0.012 | 0.021 | 92.0 | -0.012 | 0.021 | 91.5 |
| $\Sigma_{1,11}$ | 0.87 | 0.008 | 0.095 | 94.0 | 0.011 | 0.097 | 93.0 |
| $\Sigma_{1,22}$ | 0.02 | 0.001 | 0.004 | 90.5 | 0.001 | 0.004 | 95.0 |
| $\Sigma_{2,11}$ | 0.02 | 0.019 | 0.016 | 92.0 | 0.020 | 0.016 | 92.5 |
| $\Sigma_{2,22}$ | 0.91 | 0.009 | 0.084 | 97.0 | 0.008 | 0.082 | 95.0 |
| | | Classification accuracy 98.8 (98.5, 99.0) | | | Classification accuracy 98.8 (98.6, 99.0) | | |

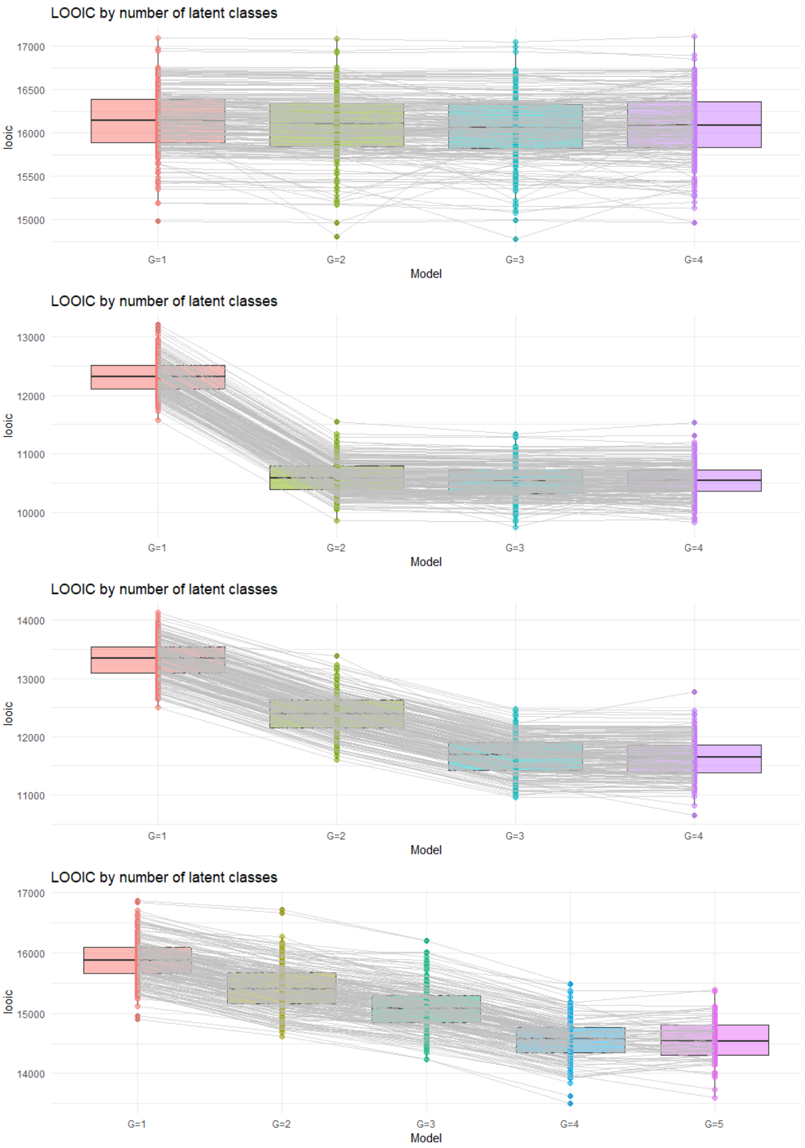


**Fig. 1**: Boxplot summary of LOOIC values (on the deviance scale) for candidate values of $G$ under four scenarios in simulation Setting I. Results are based on 200, 200, 196, and 71 data replications for Scenarios 1 ($G = 1$; top panel), 2 ($G = 2$; upper-middle panel), 3 ($G = 3$; lower-middle panel), and 4 ($G = 4$; bottom panel), respectively. Individual LOOIC values are shown as points, with lines connecting those from the same data replication across candidate models.

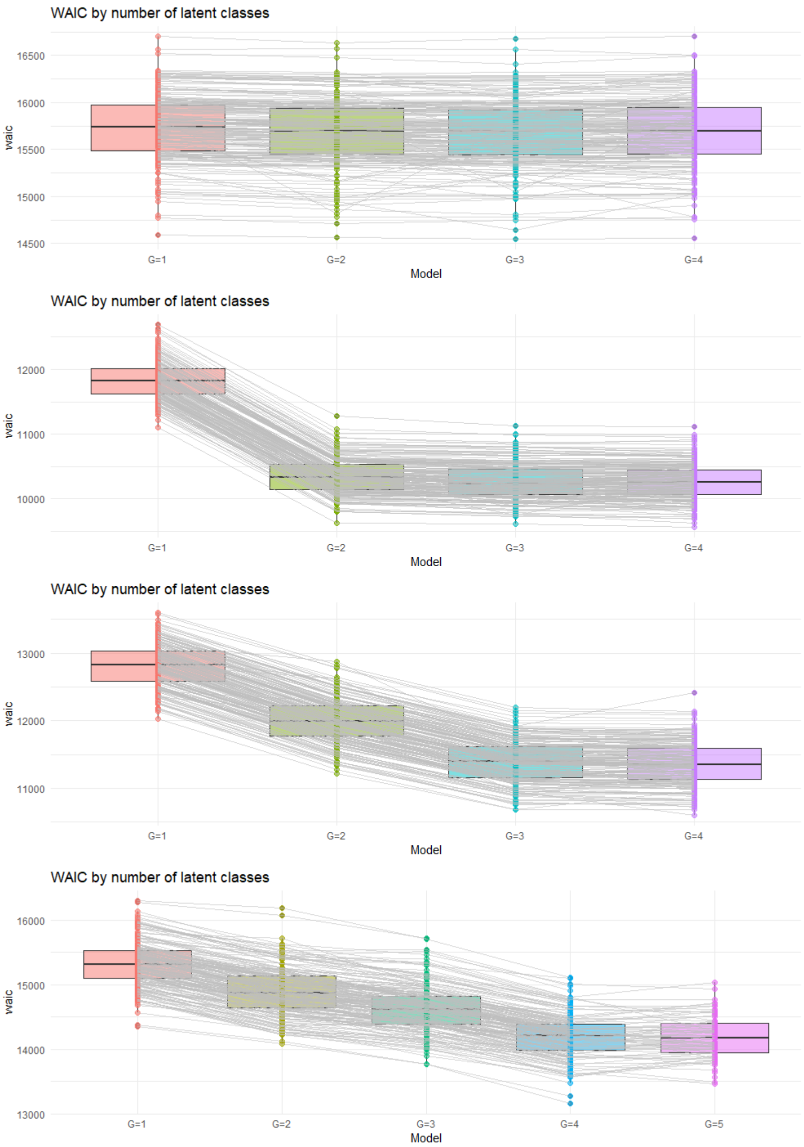


**Fig. 2**: Boxplot summary of WAIC values (on the deviance scale) for candidate values of $G$ under four scenarios in simulation Setting I. Results are based on 200, 200, 196, and 71 data replications for Scenarios 1 ($G = 1$; top panel), 2 ($G = 2$; upper-middle panel), 3 ($G = 3$; lower-middle panel), and 4 ($G = 4$; bottom panel), respectively. Individual LOOIC values are shown as points, with lines connecting those from the same data replication across candidate models.

**Table 6**: Results for simulation Setting II (Scenario 2). The settings are the same as those in Table 5.

| Parameter | True Value | Model 1 | | | Model 2 | | |
|---|---|---|---|---|---|---|---|
| | | Bias | SD | Coverage (%) | Bias | SD | Coverage (%) |
| $\beta_{1,0}$ | 8.03 | -0.006 | 0.084 | 92.5 | -0.006 | 0.085 | 93.0 |
| $\beta_{1,1}$ | -0.16 | 0.001 | 0.012 | 94.5 | 0.001 | 0.012 | 93.5 |
| $\beta_{1,2}$ | -5.86 | 0.004 | 0.120 | 92.0 | 0.005 | 0.120 | 93.5 |
| $\beta_{2,0}$ | -8.03 | 0.005 | 0.038 | 93.0 | 0.005 | 0.038 | 93.0 |
| $\beta_{2,1}$ | 0.46 | -0.003 | 0.069 | 94.0 | 0.000 | 0.071 | 94.0 |
| $\beta_{2,2}$ | 12.2 | -0.009 | 0.053 | 96.0 | -0.008 | 0.053 | 96.0 |
| $\gamma_{1,0}$ | -4.85 | -0.079 | 0.378 | 96.0 | -0.078 | 0.376 | 95.0 |
| $\gamma_{1,1}$ | -0.02 | 0.000 | 0.005 | 95.0 | 0.000 | 0.005 | 95.5 |
| $\gamma_{2,0}$ | -4.85 | -0.033 | 0.244 | 95.5 | -0.034 | 0.245 | 94.5 |
| $\gamma_{2,1}$ | 0.09 | 0.001 | 0.005 | 94.0 | 0.001 | 0.005 | 94.0 |
| $\alpha_1$ | 0.38 | 0.005 | 0.032 | 95.0 | 0.005 | 0.031 | 94.5 |
| $\alpha_2$ | 0.08 | 0.001 | 0.008 | 95.0 | 0.001 | 0.008 | 95.0 |
| $\xi_1$ | 1.8 | 0.031 | 0.112 | 95.0 | 0.030 | 0.111 | 92.5 |
| $\xi_2$ | 1.4 | 0.010 | 0.052 | 92.5 | 0.010 | 0.052 | 93.0 |
| $\sigma_1^2$ | 0.4761 | 0.001 | 0.015 | 96.5 | 0.000 | 0.015 | 96.5 |
| $\sigma_2^2$ | 0.4761 | -0.008 | 0.022 | 94.0 | -0.008 | 0.022 | 93.5 |
| $\Sigma_{1,11}$ | 0.87 | 0.012 | 0.072 | 97.0 | 0.013 | 0.072 | 97.0 |
| $\Sigma_{1,22}$ | 0.02 | 0.001 | 0.003 | 95.0 | 0.001 | 0.003 | 94.5 |
| $\Sigma_{2,11}$ | 0.02 | 0.022 | 0.018 | 90.0 | 0.022 | 0.017 | 90.5 |
| $\Sigma_{2,22}$ | 0.91 | 0.017 | 0.089 | 93.0 | 0.018 | 0.086 | 95.5 |
| | | Classification accuracy 98.7 (98.4, 98.9) | | | Classification accuracy 98.7 (98.4, 98.9) | | |

**Table 7**: Results for comparing models (under the current slope association structure) with candidate values of $G$. For each $G = k$, LOOIC and WAIC (on the deviance scale) are computed as described in Section 3.4 of the main manuscript. The $Z_{LOOIC}$ and $Z_{WAIC}$ represent the z-scores associated with the differences in LOOIC and WAIC, respectively, comparing the models with $G = k$ and $G = k + 1$. $P_{LOOIC}$ and $P_{WAIC}$ are the one-tailed p-values associated with $Z_{LOOIC}$ and $Z_{WAIC}$, respectively. $G_{eff}$ is the effective class size defined in Section 3.4, with a class proportion threshold set to 2%.

| | LOOIC | $Z_{LOOIC}$ | $P_{LOOIC}$ | WAIC | $Z_{WAIC}$ | $P_{WAIC}$ | $G_{eff}$ |
|---|---|---|---|---|---|---|---|
| G = 1 | 4311 | 7.21 | 0 | 4065 | 8.34 | 0 | 1 |
| G = 2 | 4093 | 2.30 | 0.011 | 3816 | 0.78 | 0.006 | 2 |
| G = 3 | 4043 | 0.77 | 0.220 | 3794 | 0.08 | 0.468 | 2 |
| G = 4 | 4032 | - | - | 3795 | - | - | 3 |

**Table 8**: Estimation results for the shared parameter JLCM (under the current slope association structure) with $G = 2$. Posterior means are reported as point estimates. The 95% credible intervals (CI) are based on the 2.5th and 97.5th percentiles of the posterior samples.

| **Submodel** | **Parameters** | **Estimates (Posterior Mean (95% CI))** | |
|---|---|---|---|
| | | Class 1 | Class 2 |
| Longitudinal | $\beta_{g,CEP}$ | 0.386 (0.045, 0.704) | 0.819 (0.644, 1.009) |
| | $\sigma_g^2$ | 0.334 (0.283, 0.392) | 0.214 (0.178, 0.247) |
| Time-to-event | $\alpha_g$ | -4.305 (-5.982, -2.964) | -8.390 (-11.015, -6.207) |
| | $\gamma_{g,CEP}$ | -1.535 (-3.166, -0.073) | -1.148 (-2.497, 0.085) |
| | $\gamma_{g,male}$ | -0.151 (-2.094, 1.803) | 0.649 (-1.083, 2.377) |
| | $\gamma_{g,CEP\times male}$ | 0.339 (-1.859, 2.575) | -0.329 (-2.299, 1.735) |
| Class membership | $\psi_{10}$ | -0.730 (-1.563, 0.015) | |
| | $\psi_{1,CEP}$ | 0.313 (-0.368, 1.001) | |
| | $\psi_{1,male}$ | -0.750 (-1.405, -0.072) | |

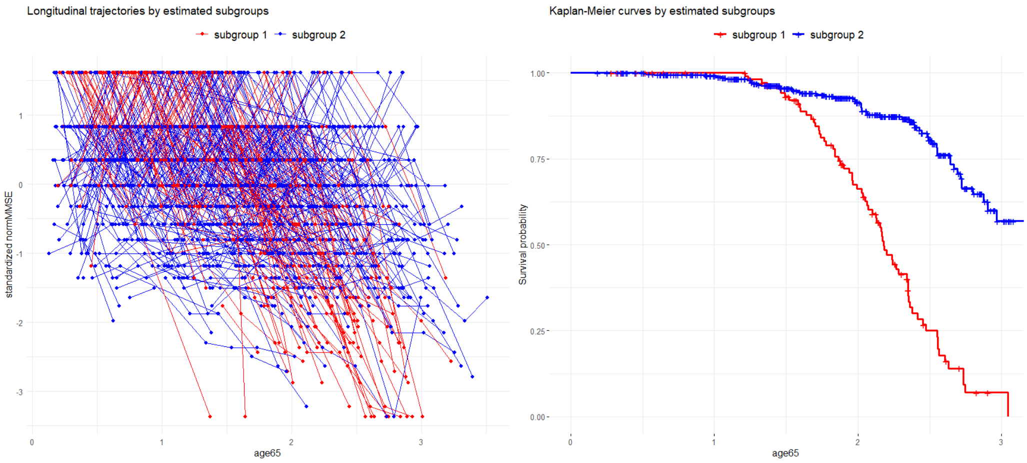


**Fig. 3**: Trajectories of standardized normMMSE and Kaplan-Meier curves by estimated subgroups with $G = 2$ (indicated by colours), based on the model with the current slope association structure.
